# Efficacy and Safety of Neoadjuvant Luteinizing Hormone-Releasing Hormone Antagonist and Tegafur-Uracil Chemohormonal Therapy for High-Risk Prostate Cancer

**DOI:** 10.3390/life13051072

**Published:** 2023-04-23

**Authors:** Fumiya Sugino, Keita Nakane, Makoto Kawase, Shota Ueda, Masayuki Tomioka, Yasumichi Takeuchi, Risa Tomioka-Inagawa, Toyohiro Yamada, Sanae Namiki, Naotaka Kumada, Shinichi Takeuchi, Kota Kawase, Daiki Kato, Manabu Takai, Koji Iinuma, Yuki Tobisawa, Takuya Koie

**Affiliations:** 1 Department of Urology, Gifu University Graduate School of Medicine, Gifu 5011194, Japan; fsugino@gifu-u.ac.jp (F.S.); keitaco@gifu-u.ac.jp (K.N.); buki2121@gifu-u.ac.jp (M.K.); risa_tom@gifu-u.ac.jp (R.T.-I.); t_yamada@gifu-u.ac.jp (T.Y.); sanae37@gifu-u.ac.jp (S.N.); kuma7821@gifu-u.ac.jp (N.K.); gallxy7@gifu-u.ac.jp (S.T.); stnf55@gifu-u.ac.jp (K.K.); andreas7@gifu-u.ac.jp (D.K.); takai_mb@gifu-u.ac.jp (M.T.); kiinuma@gifuu.ac.jp (K.I.); 2 Department of Urology, Japanese Red Cross Takayama Hospital, Takayama 5068550, Japan; jstc.mm.1215@gmail.com; 3 Department of Urology, Chuno Kosei Hospital, Seki 5013802, Japan; ch.a3200@gmail.com; 4 Department of Urology, Japanese Red Cross Gifu Hospital, Gifu 5028511, Japan; yha2s5u18@gmail.com; 5 Department of Urology, Hirosaki University Graduate School of Medicine, Hirosaki 0358562, Japan

**Keywords:** high-risk prostate cancer, robot-assisted radical prostatectomy, neoadjuvant chemohormonal therapy, luteinizing hormone-releasing hormone antagonist, tegafur-uracil

## Abstract

Background: This retrospective single-center cohort study evaluated the efficacy and safety of a combination of neoadjuvant luteinizing hormone-releasing hormone (LHRH) antagonist and tegafur-uracil (UFT) therapy (NCHT) and investigated the medical records of patients with high-risk PCa who underwent robot-assisted radical prostatectomy (RARP). The therapy was followed by RARP for high-risk PCa. Materials and Methods: The enrolled patients were divided into two groups: low-intermediate-risk PCa patients who underwent RARP without neoadjuvant therapy (non-high-risk) and those who underwent NCHT followed by RARP (high-risk group). This study enrolled 227 patients (126: non-high-risk and 101: high-risk group). Patients in the high-risk-group had high-grade cancer compared to those in the non-high-risk-group. Results: At the median follow-up period of 12.0 months, there were no PCa deaths; two patients (0.9%) died of other causes. Twenty patients developed biochemical recurrence (BCR); the median time until BCR was 9.9 months after surgery. The 2-year biochemical recurrence-free survival rates were 94.2% and 91.1% in the non-high-risk and high-risk-group, respectively (*p* = 0.465). Grade ≥3 NCHT-related adverse events developed in nine patients (8.9%). Conclusions: This study indicates that combining neoadjuvant LHRH antagonists and UFT followed by RARP may improve oncological outcomes in patients with high-risk PCa.

## 1. Introduction

According to the National Comprehensive Cancer Network (NCCN) guidelines, high-risk prostate cancer (PCa) is characterized by initial prostate-specific antigen levels (PSA) >20 ng/mL, Gleason grade (GG) ≥4 according to the International Society of Urologic Pathology (ISUP) 2014 guidelines [1], or clinical T stage ≥T3 [2]. It is widely recognized that high-risk PCa is associated with a higher likelihood of progressive, symptomatic disease or death due to PCa [3]. Treatment options for high-risk PCa include radiation therapy (RT) with or without androgen deprivation therapy (ADT), radical prostatectomy (RP), RP with neoadjuvant or adjuvant therapy, and ADT monotherapy [4,5]. Although the optimal treatment for these patients remains controversial, with no clear evidence from randomized trials, the majority of patients with high-risk PCa are treated with RT and ADT as definitive curative therapy [6,7]. In a landmark study supporting the efficacy of this treatment modality, the combination of ADT and external-beam radiation RT (EBRT) for patients with locally advanced PCa resulted in a 5-year cancer-specific survival (CSS) rate of 94% compared with 79% for EBRT alone [8]. Therefore, the proportion of patients with high-risk PCa undergoing RP has halved to less than 10%, whereas the proportion receiving EBRT with ADT has increased from 40% to 60% over the past 20 years [9]. However, ADT must be administered for 2–3 years to achieve maximum benefit. This raises the issue of ADT-related metabolic abnormalities that may accelerate the progression of PCa, increase insulin resistance, lead to the development of dyslipidemia and sarcopenic obesity, and increase the risk of cardiovascular disease and non-tumor mortality [10,11].

Surgeon and hospital volumes are considered important factors because outcomes after RP are highly dependent on surgical and pathologic features such as positive surgical margins (PSM), extracapsular extension, and seminal vesicle invasion. Several recent studies have reported that RP could improve oncologic outcomes for patients with high-risk PCa, including overall survival (OS) and CSS [4,12,13,14]. Thus, various neoadjuvant therapies prior to RP aim to improve surgical outcomes prior to RP. Studies have reported the efficacy of chemohormonal therapy (NCHT) prior to RP in patients with high-risk PCa because neoadjuvant hormone therapy (NHT) did not improve oncologic outcomes [15,16,17]. In our previous studies, neoadjuvant ADT plus estramustine phosphate (EMP) therapy improved biochemical recurrence in patients [18,19,20]. However, NCHT is not widely used because of concerns about adverse events caused by anticancer agents and the risk of deep vein thrombosis or gynecomastia caused by estrogen [15,16,17,18,19,20,21].

Tegafur-uracil (UFT) is an oral cytotoxic prodrug of 5-fluorouracil (5-FU) that contains tegafur and uracil in a 1:4 molar ratio [22,23]. Tegafur is metabolized in the liver to 5-FU, and uracil prevents 5-FU degeneration [22]. Adjuvant chemotherapy with UFT has been demonstrated to be effective against several types of cancer [22]. Uracil competitively inhibits 5-FU metabolism and prevents its rapid degradation, resulting in tumor-selective prolonged 5-FU activity [23]. The efficacy and safety of UFT-containing regimens have been reported for various malignant neoplasms, including lung, breast, gastric, and castration-resistant PCa (CRPC) [22,23,24]. The usefulness of UFT for treating CRPC has been demonstrated in late-line administration and combination with other anticancer agents [22,23,24]. Therefore, we focused on the UFT that is easy to administer and effective for CRPC. In addition, we hypothesized that its use as an NCHT for high-risk PCa might improve oncological outcomes.

We aimed to evaluate the efficacy and safety of a combination of neoadjuvant luteinizing hormone-releasing hormone (LHRH) antagonist and UFT therapy (LHRH + UFT) followed by robot-assisted RP (RARP) for high-risk PCa compared to those with low- and intermediate-risk PCa who underwent RARP alone.

## 2. Materials and Methods

### 2.1. Patient Selection

This study was approved by the Institutional Review Board of Gifu University (approval number: 2018-213). This study obtained consent for all enrolled patients, including low-, intermediate-, and high-risk PCa undergoing RARP. The details of this study can be found at https://www.med.gifu-u.ac.jp/visitors/disclosure/docs/2018-212.pdf (accessed on 3 December 2022).

This single-center non-randomized prospective study investigated our institution’s medical records of 285 patients with PCa who underwent RARP between September 2017 and September 2022. Participants had histologically confirmed PCa, no lymph node metastases or distant metastases on radiological examinations, Eastern Cooperative Oncology Group performance status of 0–1 [25], adequate bone marrow function (absolute neutrophil count > 1500/m^3^, platelet count > 100,000/m^3^), adequate renal function (creatinine <2.0 mg/dL and/or creatinine clearance >40 mL/min), and adequate liver function (total bilirubin < 1.5 mg/dL). The following clinical data of enrolled patients were collected: age, height, weight, PSA level, prostate volume, biopsy GG, clinical stage, and risk stratification using the NCCN criteria [2]. In addition, the following surgical outcomes and pathological characteristics were recorded: console time (time from start to end of RP using the robotic surgical system), estimated blood loss, GG of the surgical specimens, tumor (T) and node (N) stages of the surgical specimens, and presence of positive resection margins. Tumors were staged according to the American Joint Committee on Cancer 8th Edition “Cancer Staging Manual” [26]. No data were collected in this study to determine whether the enrolled patients underwent magnetic resonance imaging evaluation prior to prostate biopsy, lymphovascular invasion, and seminal vesicle involvement. Based on the ISUP 2014 guidelines [1], the GGs of the biopsy and surgical specimens were evaluated and classified into five groups.

According to the NCCN criteria [2], the enrolled patients were divided into two groups: those with low- or intermediate-risk PCa who underwent RARP alone without neoadjuvant therapy (non-high-risk) and those who underwent NCHT followed by RARP (high-risk group). We compared the surgical and oncological outcomes between the patients from the non-high-risk and high-risk group who underwent RARP at our institution.

### 2.2. Treatment

In the high-risk group, all patients received an LHRH antagonist (degarelix with a starting dose of 240 mg for one month and a monthly maintenance dose of 80 mg after that) and UFT (300 mg/day) for at least three months before RARP. All patients underwent computed tomography and magnetic resonance imaging prior to RARP, confirming the absence of levator muscle invasion, locoregional and distant lymph node involvement, and distant metastases. All enrolled in the study did not undergo pelvic lymph node dissection (PLND) for the reasons we have previously reported [27,28].

### 2.3. Pathological Analysis

All prostatectomy specimens were assessed by whole-mount staining according to the ISUP guidelines [1]. The pathology of the prostatic apex was evaluated by cutting it perpendicular to the prostatic urethra. The end of the bladder neck was cut from the excised specimen in a conical shape and cut perpendicular to prepare the pathology specimen. The remaining prostate tissue was sectioned at 3–5 mm intervals along a plane perpendicular to the urethral axis for pathological evaluation.

### 2.4. Follow-Up Schedule

Patients in the study were evaluated for BCR by measuring their serum PSA and testosterone levels every three months after RARP for the first 2 years and every 6 months for 5 years thereafter. BCR was defined as a postoperative increase in serum PSA level of > 0.2 ng/mL with a second confirmed PSA of >0.2 ng/mL [29]. If the postoperative PSA level never fell below 0.2 ng/mL, the date of RARP was considered to be the time when BCR occurred. PSA values were measured by Cobas (Roche diagnostics; Basel, Switzerland).

### 2.5. Statistical Analysis

The endpoint of this study was biochemical recurrence-free survival (BRFS). JMP 14 software (SAS Institute Inc., Cary, NC, USA) was used for data analysis. BRFS after RARP was assessed using the Kaplan–Meier method. The relationship between BCR and subgroup classification was analyzed using the log-rank test. *p* values of < 0.05 were considered statistically significant.

## 3. Results

### 3.1. Patient Characteristics

Preoperative patient demographic data are shown in Table 1. In total, 227 patients were enrolled in this study. One patient (0.8%) who received ADT before RARP in the non-high-risk, 20 patients (12.8%) who received ADT in the RARP group, and 35 (22.4%) who received ADT before RARP in the high-risk group were excluded from the study.

### 3.2. Comparison of Clinical and Pathological Covariates between Two Groups

Surgical and pathological outcomes were compared between the two groups (Table 2). Although operation time was significantly shorter in the high-risk group, the number of organ-confined PCa was significantly higher in the non-high-risk group. However, there was no significant difference in PSM between the two groups.

### 3.3. Oncological Outcomes

**Figure 1:**
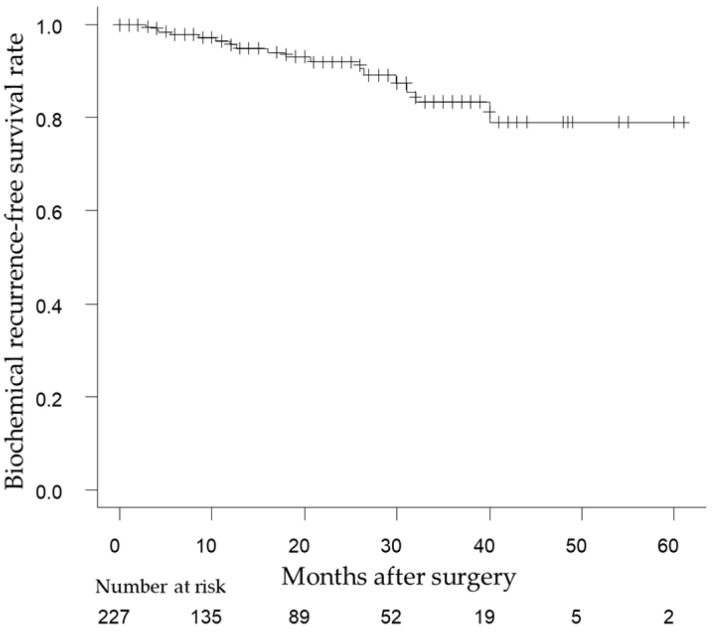
Analysis of biochemical recurrence-free survival (BRFS) using the Kaplan–Meier method in all patients receiving RARP of this study. The 1- and 2-year BRFS rates in all patients were 95.8% and 92.0%, respectively.

The median follow-up period was 17.0 months (interquartile range [IQR], 6–30 months). During the follow-up period, there were no PCa deaths among the enrolled patients, but two (0.9%) died of other causes (details unknown). Twenty patients developed BCR; the median time until BCR was recognized as 16.0 months after surgery (IQR, 8.5–29.9 months). The 1- and 2-year BRFS rates in all patients were 95.8% and 92.0%, respectively (Figure 1).

**Figure 2:**
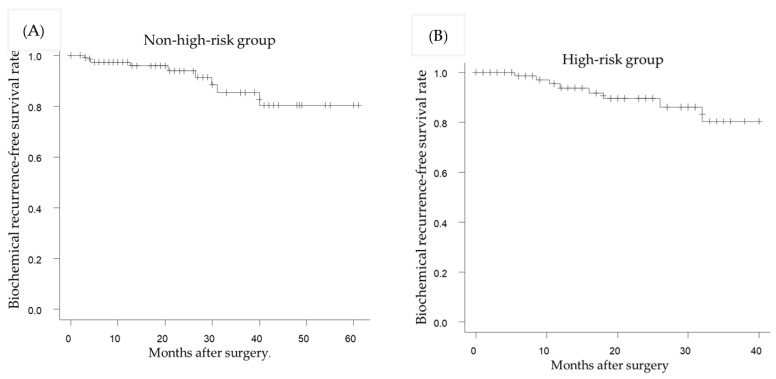
Kaplan–Meier estimate of the biochemical recurrence after robot-assisted radical cystectomy in patients with or without receiving neoadjuvant chemohormonal therapy. The 2-year biochemical recurrence-free survival rates were 93.9% and 89.5% in the non-high-risk group (**A**) and high-risk group (**B**), respectively (*p* = 0.542).

The 1- and 2-year BRFS rates in the non-high-risk group were 97.4% and 93.9%, respectively (Figure 2A). The 1- and 2-year BRFS rates in the high-risk group were 93.7% and 89.5%, respectively (Figure 2B). There was no significant BRFS association between the two groups (*p* = 0.542).

### 3.4. Safety of NCHT

NCHT-related deaths were also reported during the study period. The grade ≥3 NCHT-related adverse events were liver disorder in seven patients (6.9%), a rash with the liver disorder in one (0.9%), and anorexia with liver disorder in one (0.9%) according to the Clavien–Dindo classification [30]. Six patients (5.9%) received a reduced dose of UFT, and three (3.0%) discontinued NCHT. All adverse events occurred within 3 months of the administration of NCHT. All the patients who developed adverse events improved with RARP.

## 4. Discussion

According to EAU guideline, there is no current consensus on the optimal treatment for high-risk PCa. The oncological outcomes associated with high-risk PCa are heterogenous [4,5,6,7,31,32,33]. For the past 20 years, RT plus ADT has been the mainstay of treatment for high-risk PCa [6,7,8,9]. Additionally, several recent studies have reported the usefulness of trimodality therapy for high-risk PCa [7,31,34]. Kishan et al. [7] reported adjusted 5-year PCa-specific mortality rates of 12% for RP, 13% for EBRT, and 3% for EBRT plus brachytherapy (BT). EBRT plus BT was significantly associated with lower PCa-specific mortality than either RP or EBRT alone [7]. According to the incidence of distant metastases, EBRT + BT had a significantly lower rate compared to the other two treatments [7]. In our previous study, the 10-year BRFS rate for patients with high-risk PCa who received EBRT, BT, and ADT during a 24-month period was 93.4% [34]. Indeed, the combination of RT and ADT therapy for high-risk PCa has been reported to have relatively favorable oncological outcomes, with 5- and 10-year CSS rates of 94% and 84%, respectively [6]. Lu et al. [31] compared the oncological outcomes of RT plus ADT and RP for high-risk PCa in a propensity score-matching study. Multivariate Cox regression analysis showed that treatment with RT + ADT significantly reduced the risk of BCR compared to RP (hazard ratio [HR] 0.162, *p* < 0.001). However, there was no significant difference in the risk of local recurrence, distant metastasis, or OS between the two treatment modalities (*p* = 0.470, *p* = 0.268, *p* = 0.509, respectively) [28]. Therefore, it is necessary to determine which treatment should be chosen for high-risk PCa when considering OS as an endpoint.

A Swedish 15-year observational study of 34,515 patients with PCa reported that RP significantly reduced oncological outcomes, such as OS and CSS, compared to RT, even in patients with high-risk PCa [12]. Although the results of this study may have led to a gradual re-evaluation of the usefulness of RP for high-risk Ca, it has long been recognized that RP is likely to contribute to improve OS. When 68,665 patients with localized PCa who underwent RP or RT were identified from the Surveillance, Epidemiology, and End Results (SEER) database using propensity score matching, the 10-year cancer-specific mortality rate among patients with high-risk PCa was 6.8% for RP and 8% and 11.5% for RT (*p* < 0.001) [34]. Tewari et al. [10] reported a cancer-specific mortality risk of 13.4% for RP, 16.8% for RT, and 43% for observation in 453 patients with GG 4 PCa after a median follow-up of over 4 years. Although there was no significant difference between RP and RT, patients who received RP had a relatively longer CSS compared to those who received RT among patients with high-risk PCa (*p* = 0.053) [10]. A recent study comprising 9114 patients with high-risk PCa with a median follow-up of 4.7 months using the SEER database showed that patients who received RP had significantly better OS than those who received EBRT and EBRT + BT (HR: 3.36, *p* < 0.001; HR 2.15, *p* = 0.002, respectively) [13]. In addition, there was no statistical difference in PCa-specific mortality between RP and EBRT plus BT (HR: 1.32, *p* = 0.485); however, patients who received EBRT had poor OS (*p* < 0.05) [13]. In contrast, oligometastatic PCa, having a limited number of metastatic foci in a specific location, is attracting attention as an early stage of multistage metastasis [35,36]. Therefore, as with non-metastatic PCa, efficient and effective treatment of all tumor sites, including cytoreductive RP, may improve the oncological outcomes in some patients with oligometastatic PCa [35,36]. Several hypotheses have been proposed, although the role of local therapy in high-grade or high-risk PCa remains unclear [37,38,39]. Kim et al. reported that primary tumors might serve as a source of circulating tumor cells with the potential for “self-seeding [37]”. It has also been suggested that the growth of distant metastases may be stimulated and maintained by compounds secreted by the primary tumor [38]. Based on these theories, RP in patients with high-risk or high-grade PCa may potentially reduce the initiation and progression of distant metastases, making it more likely that RP contributes to improved oncological outcomes compared with other treatment modalities.

Although several guidelines recommend that surgery be part of a comprehensive treatment for high-risk PCa [2,4], pathological findings suggest that a relatively high proportion of PSM may not be cured by RP and consequently require adjuvant therapy [38]. Therefore, neoadjuvant therapy is administered to improve surgical and oncologic outcomes in patients with high-risk PCa [15,16,40,41,42]. NHT prior to RP has been tried in combination with cytotoxic agents as it was found to increase the negative surgical margin rate but not prevent BCR [15,16,17,18,19,20]. Narita et al. [15] reported the utility of neoadjuvant ADT, docetaxel (DOC), and EMP followed by RP in patients with high-risk PCa compared with surgery alone. The 5-year BRFS rate was 60.1%, with a median follow-up period of 42.5 months [15]. Although 10.0% achieved a complete pathologic response, 13.3% developed grade 3 or higher NCHT-related complications [15]. A Phase 2 study of neoadjuvant DOC and ADT for high-risk PCa showed that 27% of patients had PSM and four had lymph node metastases [16]. At a median observation period of 42.7 months, BCR was observed in 30% of the patients, suggesting that it was not likely to be an effective treatment [16]. Recently, Ravi et al. [42] reported the efficacy of NHT with second-generation antiandrogens before RP in high-risk patients with PCa. After inverse probability of treatment weighting, time to BCR (HR = 0.25) and metastasis-free survival (HR = 0.26) were significantly longer in patients receiving NHT with second-generation antiandrogens than in those receiving RP alone [41]. In our previous studies, neoadjuvant LHRH plus EMP followed by RP was significantly associated with oncological outcomes, such as BRFS and OS, compared to RP alone for high-risk PCa [18,19,20]. However, preoperative treatment regimens that include EMP are not widespread because of concerns regarding the risk of deep vein thrombosis and gynecomastia caused by estrogen [21]. Therefore, neoadjuvant therapy for high-risk PCa may change, and more patients with high-risk PCa will opt for RARP with neoadjuvant therapy. Comparing the results of treatment with our previous study of LHRH + EMP, the BRFS of high-risk PCa was almost comparable, although the observation periods were different [20]. On the other hand, the PSM rate in this study was 18.8%, and that of ADT + EMP was 8.0%, which tended to be slightly higher in the group enrolled in this study [20]. However, the PSM rate of the LHRH + EMP group was 26.8% when examined with the data before propensity score matching [20], therefore, the PSM of the LHRH + UFT group was considered to be reasonable. The NCCN guideline recommend PLND for patients with intermediate- and high-risk PCa [2]. In a study in which PLND was associated with an improved BCR, a 2-year BRFS rate of 93% was reported in 439 patients who underwent RARP with PLND, although the median follow-up period was only 16 months [43]. According to a multicenter retrospective cohort study of 3195 patients with PCa who underwent RARP at nine institutions in Japan, the 2-year BRFS rates were 95.8% in patients who underwent RARP without PLND and 94.3% in those with PLND (*p* = 0.855) [28]. In this study, the BRFS rate in the ADT + UFT group was also similar to that in the non-high-risk PCa group and the RARP + PLND group [28]. Since this study was not a randomized controlled trial and the observation period was relatively short, the pros and cons of PLND need to be further discussed.

It has been reported that different methods of PSA measurement yield different results [44]. Therefore, it is necessary to determine whether the bias between measurement methods is within an acceptable range [44]. In fact, the treatment method of choice for PSA varies greatly depending on its value, so it is important to evaluate the impact of inter-method bias on clinical outcomes [45]. In this study, PSA could be measured in a similar manner due to the short observation period. However, it is important to keep in mind that differences in PSA measurement methods may affect the results and the choice of treatment. Ferraro et al. [46] defined PSA thresholds by progression using a calibrated risk prediction model to individualize the diagnosis by the Roche assay. In patients <65 years, PSA values >5.7 μg/L had a positive predictive value (PPV) of 35.9%, and ≤4.1 μg/L and ≤4.9 μg/L had negative predictive values (NPV) of 95.1% and 97.5%, respectively. On the other hand, in patients ≥65 years, a PSA of ≥5.3 μg/L resulted in a PPV of about 50%, while a PSA of <3.7 μg/L resulted in a NPV of 88.8%, suggesting that advanced PCa could be excluded [46]. Therefore, it was possible that there might be differences in the malignancy and progression of PCa depending on age and PSA levels.

In this study, we reported that neoadjuvant LHRH antagonist and UFT combination therapy for high-risk PCa was similar to the BCR of low- and intermediate-risk PCa in patients who underwent RARP. Selecting UFT as a neoadjuvant treatment for high-risk PCa was based on the results of subsequent clinical studies [22,23,24]. Takahashi et al. [22] conducted a multicenter prospective randomized phase II trial to evaluate the efficacy and safety of ADT + UFT versus ADT alone for CRPC. ADT plus UFT had a significantly longer time to PSA progression compared to ADT alone [22]. In patients with low expression of thymidylate synthase or high expression of orotate phosphoribosyltransferase, ADT plus UFT demonstrated a higher PSA response rate than ADT alone (*p* = 0.019 and *p* = 0.041, respectively) [22]. Thus, ADT + UFT was more effective and better tolerated against CRPC than ADT alone [22]. Hayakawa et al. [23] showed that UFT administration as a fourth-line treatment was well-tolerated and somewhat effective in patients with CRPC who had already received ADT, alternative androgen therapy, and EMP. Another study reported that 63% of patients with CRPC had a PSA reduction of 50% or greater after oral combination therapy with dexamethasone, UFT, and cyclophosphamide, with a median time to progression of 7.2 months [24]. Indeed, the possibility that an increase or decrease in PSA does not always accurately reflect the therapeutic effect should be considered. However, neoadjuvant ADT + UFT followed by RARP could achieve suppression of BCR in approximately 90% of the patients with high-risk PCa. Therefore, NCHT may have a certain anti-cancer effect on high-risk PCa.

Neoadjuvant therapy with an LHRH antagonist and UFT is considered an effective treatment for patients with high-risk prostate cancer. However, approximately 10% of the patients had grade 3 adverse events, suggesting that treatment should be conducted under close observation.

This study has several limitations; therefore, caution is required when interpreting the results. First, as this was a single-center retrospective study, there is a possibility of bias in the results. Second, the small number of patients enrolled and the relatively short follow-up period of this study may not accurately reflect the efficacy of neoadjuvant LHRH antagonists plus UFT followed by RARP in patients with high-risk PCa. Hence, long-term follow-up is necessary to verify the treatment efficacy. Third, because lymph node dissection was not performed at the time of RARP, the efficacy of this treatment in patients with possible lymph node metastases has not been confirmed. Fourth, this was a single-arm study to evaluate the efficacy of neoadjuvant LHRH + UFT for high-risk PCa, and was not randomized. Additionally, because a very small number of patients with high-risk PCa receiving RARP alone were identified in our institute, the non-high-risk group treated at the same time was used as a comparison group. BRFS was similar in both groups, although the control group was likely inappropriate. Finally, a multicenter prospective study is required to demonstrate the efficacy of this treatment.

## 5. Conclusions

This study showed that the combination of neoadjuvant LHRH antagonists and UFT followed by RARP may improve oncological outcomes in patients with high-risk PCa. Further prospective randomized trials and long-term evaluations are needed to validate the oncologic outcomes in patients treated with neoadjuvant chemohormonal therapy.

## Figures and Tables

**Table 1:** Clinical covariates of the enrolled patients.

Variables	Non-High-Risk Group	High-Risk Group	*p*
	N = 126	N = 101	
Age (year, median, IQR)	70 (67.0–73.0)	72.0 (68.0–74.0)	0.055
Initial PSA (ng/mL, median, IQR)	6.50 (4.90–8.95)	9.88 (5.80–12.87)	<0.001
Prostate volume (cc, median, IQR)	35.0 (27.7–47.0)	28.0 (20.0–39.3)	<0.001
Biopsy Gleason Grade (number, %)			<0.001
1	36 (28.6)	0	
2	48 (38.1)	8 (7.9)	
3	42 (33.3)	8 (7.9)	
4	0	50 (49.5)	
5	0	35 (34.7)	
Clinical T stage (number, %)			0.003
T1c	18 (14.2)	3 (3.0)	
T2a	92 (73.0)	41 (40.6)	
T2b	4 (3.2)	12 (11.9)	
T2c	12 (9.5)	23 (22.8)	
T3a	0	17 (16.8)	
T3b	0	5 (5.0)	
NCCN risk classification (number, %)			<0.001
Low	27 (21.4)	0	
Intermediate	99 (78.6)	0	
High	0	101 (100.0)	
Follow-up period (months, median, IQR)	17.5 (6.2–35.2)	12.0 (5.0–29.0)	>0.999

IQR: interquartile range; PSA: prostate-specific antigen.

**Table 2:** Surgical and pathological outcomes in the enrolled patients.

Variables	Non-High-Risk Group	High-Risk Group	*p*
	N = 126	N = 101	
Console time (minutes, median, IQR)	131 (111–168)	113 (87–132)	<0.001
Estimated blood loss (mL, median, IQR)	27.5 (5.0–75.0)	25.0 (5.0–75.0)	0.790
Pathological Gleason Grade (number, %)			<0.001
pT0	0	2 (2.0)	
1	9 (7.1)	1 (1.0)	
2	65 (51.6)	19 (18.8)	
3	41 (32.5)	13 (9.9)	
4	4 (3.2)	23 (12.9)	
5	7 (5.6)	43 (42.6)	
Pathological T stage (number, %)			0.003
pT0	0	2 (2.0)	
pT2	108 (85.7)	72 (71.2)	
pT3	18 (14.3)	27 (26.8)	
Positive surgical margin (number, %)	23 (18.3)	19 (18.8)	>0.999

IQR: interquartile range.

## Data Availability

The data presented in this study are available on request from the corresponding author. The data are not publicly available due to privacy and ethical reasons.
